# FHL1 activates myostatin signalling in skeletal muscle and promotes atrophy

**DOI:** 10.1016/j.fob.2015.08.011

**Published:** 2015-09-01

**Authors:** Jen Y. Lee, Dede Lori, Dominic J. Wells, Paul R. Kemp

**Affiliations:** a Section of Molecular Medicine, National Heart and Lung Institute, Imperial College London, South Kensington Campus, London SW7 2AZ, UK; b Comparative Biomedical Sciences, Royal Veterinary College, Royal College Street, London NW1 0TU, UK

**Keywords:** FHL1, four and a half LIM domain protein 1, COPD, chronic obstructive pulmonary disease, TGF-β, transforming growth factor beta, MHC, myosin heavy chain, GDF-15, growth and differentiation factor 15, PAI-1, plasminogen activator inhibitor 1, TA, tibialis anterior, VEGF-C, vascular endothelial growth factor C, Muscle wasting, Fibre type, FHL1, Myostatin, Mouse

## Abstract

Myostatin is a TGFβ family ligand that reduces muscle mass. In cancer cells, TGFβ signalling is increased by the protein FHL1. Consequently, FHL1 may promote signalling by myostatin. We therefore tested the ability of FHL1 to regulate myostatin function. FHL1 increased the myostatin activity on a SMAD reporter and increased myostatin dependent myotube wasting. In mice, independent expression of myostatin reduced fibre diameter whereas FHL1 increased fibre diameter, both consistent with previously identified effects of these proteins. However, co-expression of FHL1 and myostatin reduced fibre diameter to a greater extent than myostatin alone. Together, these data suggest that the expression of FHL1 may exacerbate muscle wasting under the appropriate conditions.

## Highlights

• Myostatin signals via SMADs to promote muscle wasting.
• FHL1 normally promotes hypertrophy but can activate SMAD signalling.
• FHL1 promoted myostatin signalling *in vitro*.
• FHL1 promoted hypertrophy in the absence of myostatin but atrophy in its presence.

## 1 Introduction

Voluntary movement is essential for a normal healthy life and the performance of daily activities. Such movement requires a sufficient quantity of skeletal muscle especially in the locomotor muscles and the appropriate proportions of the different fibre types. Different fibre-types have distinct rates of contraction and abilities to endure activity. The overall phenotype of a muscle is related to the relative proportions of the different fibres it contains. Muscle phenotype is plastic and the size and proportion of the individual fibres can change dependent on a number of factors including physical activity.

Changes in muscle mass and phenotype are important aspects of a number of chronic diseases such as chronic obstructive pulmonary disease (COPD), heart failure and cancer and have prognostic ability. Indeed exercise capacity and strength are better predictors of survival in patients with COPD than standard measures of pulmonary function [1]. Muscle mass is also lost in ageing and there is a marked change in phenotype again with prognostic implications [2,3]. Consequently the factors that affect muscle mass are being intensively studied.

Not all fibres atrophy at the same rate and a number of studies have shown that type II fibres are more likely to atrophy than type I fibres in diseases as varied as COPD [4], heart failure [5] and osteoarthritis [6] as well as in normal human ageing [7]. As there is a shift towards type II fibres in the quadriceps muscles in chronic disease, this increased sensitivity of type II fibres to atrophy is likely to contribute to accelerated wasting. Under some conditions (e.g. starvation) where muscle acts as an emergency fuel store, this response may be important; however, in chronic disease it is likely to be detrimental.

Myostatin is one factor likely to be involved in the increased susceptibility of type II fibres to atrophy. This growth regulator is a member of the transforming growth factor-β (TGF-β) family that was identified from natural mutations in animals with a double muscled phenotype [8,9]. Germ-line deletion of the myostatin gene from mice resulted in a similar hypermuscular phenotype suggesting that myostatin is an inhibitor of muscle growth. Deletion of myostatin also increased the proportion of the fastest type IIB fibres suggesting that the major effects of myostatin were on this fibre type [10,11]. Furthermore myostatin expression is highest in type IIB fibres [12], is elevated in response to hind limb suspension and is a target for the type I fibre restricted microRNA, miR-499. Indeed it has been shown that increased myostatin mRNA and protein are associated with type II muscle atrophy [13].

Myostatin signals by binding to an activin IIB/alk4/5 receptor complex promoting the phosphorylation of SMAD-2/3. Consequently factors that modify SMAD-2/3 phosphorylation are likely to alter myostatin signalling. One protein that activates SMAD proteins is the four and a half LIM domain protein FHL1 which binds to CKδ and promotes SMAD phosphorylation [14]. In muscle cells such an activity would promote muscle atrophy but under normal conditions FHL1 appears to promote hypertrophy. For example, over-expression of FHL1 leads to muscle hypertrophy [15] and patients with mutations in FHL1 have a range of myopathic conditions including X-linked myopathy with postural muscle atrophy (XMPMA) [16]. However, a number of studies have shown that FHL1 can associate with atrophy. For example, denervation in mice increases FHLI [17], long-term training in humans reduces FHL1 expression [18] and we observed that FHL1 was associated with weakness in COPD patients [19]. These observations raise the possibility FHL1 potentiates myostatin signalling in muscle cells so contributes to muscle wasting under a subset of conditions. Consequently in this study we determined the effect of FHL1 on SMAD reporter gene expression in response to myostatin, as well as the effect of FHL1 on myostatin induced myotube wasting. Finally we used electroporation of the *tibialis anterior* (TA) in mice to determine the effect of FHL1 on myostatin induced muscle wasting *in vivo.*

## 2 Materials and methods

### 2.1 Cloning

Full-length murine myostatin and FHL1 were sub-cloned into pGEMT by PCR from image clones (Source Bioscience) then shuttled into empty pCAGGS expression vector (containing a CMV-enhancer and chicken β-actin promoter) [20] and sequenced. Large-scale plasmid preparations were carried out using the EndoFree Mega kit (Qiagen) according to manufacturer’s instructions and plasmids were eluted in sterile dH_2_0.

### 2.2 Cell culture, luciferase reporter and myotube diameter assays

For luciferase experiments, C2C12 myoblasts were cultured and transfected using lipofectamine as described in [21] scaled for culture in 24 well plates. A total of 0.4 μg DNA was transfected per well in total: 0.2 μg of (CAGA)_12_-luciferase plasmid [22], 0.1 μg of pRLTK plasmid, 0.1 μg of either FHL1-pcDNA or 0.1 μg pcDNA as the control. After transfection the cells were cultured in DMEM supplemented with 10% (v/v) FBS. Twenty-four hours later, cells were serum starved for 7 h then washed and treated with serum-free DMEM containing either TGF-β1 (Insight Biotechnology) or Myostatin (PromoKine) for 16 h. Luciferase activity was assayed using the Dual-Luciferase® Reporter Assay System (Promega) according to the manufacturer’s instructions. Firefly luciferase activity was normalised to Renilla luciferase activity to account for transfection efficiency. All data were normalised to the mean of the pcDNA untreated (0 ng/mL TGF-β) group for that set of transfections.

For myotube diameter measurements, C2C12 myoblasts were cultured and transfected as described in [23] with a total of 2 μg DNA consisting of 0.5 μg pCAGGS-GFP [24]and either 1.5 μg FHL1-pcDNA or 1.5 μg pcDNA. Thus, myoblasts that were successfully transfected with pCAGGS-GFP would also be transfected with the FHL1 expression vector and allow identification of fluorescent ‘transfected’ myotubes. After 4.5 h incubation, media was replaced with fresh DMEM + 10% FBS and the cells returned to the incubator. The medium was replaced every 2 days and allowed to become confluent (approximately 3 days). The media was replaced with DMEM supplemented with 2% horse serum (differentiation medium) to differentiate myoblasts into myotubes for 7 days, with fresh replacement every 2 days. Myotube formation was confirmed by the presence of multinucleate cells and parallel experiments looking at the expression of myogenic regulatory factors and MHCs showed an increase in the expression of all MHCs with an increase in myogenin expression consistent with previous observations [25,26] (data not shown). After 7 days, the media was then replaced with differentiation medium supplemented with 20 ng/mL myostatin (PromoKine) or vehicle control (0.1% BSA and 20 mM HCl). After 48 and 96 h of exposure, fluorescent myotubes were identified in randomly selected views at 10× magnification and captured using a Hamamatsu C4742-95-12ERG camera attached to a Zeiss Axiovert 200 microscope with the filter set 470 nm/40 nm. Average myotube diameter was ascertained by measuring the shortest distance across the myotube at five points along the length of fluorescent myotubes using Image J.

### 2.3 Electroporation

Mouse experiments were approved by the Royal Veterinary College Ethical Review Process (ERP-A-2010-WS01) and were licensed by the UK Secretary of State for the Home Office under Project License PPL 70/6797. Twelve female CD1 mice (7.5 weeks old) were anaesthetised with Hypnorm (VetaPharma) and Hypnovel (Roche), both lower legs were shaved and 10U (25 μl) of bovine hyaluronidase (Sigma) was injected percutaneously into each TA to increase transfection efficiency [27,28]. Mice were allowed to partially recover at 37 °C and after 1.5 h they were re-anaesthetised using 5% isofluorane and maintained at 2% isofluorane. The TA muscles were injected with 25 μl of the appropriate plasmid at 1 mg/mL. Immediately following the plasmid injection electro-conductive cream was applied to electrodes which were placed either side of TA, separated by approximately 5 mm. Electroporation was performed using 10 pulses of 85 V each for 20 ms, at a frequency of 1 Hz.

Following electroporation, mice recovered and were left for 2 weeks, after which the mice were sacrificed and TA muscles were harvested and placed upright onto small pieces of cork with a small amount of OCT at the bottom to fix the bottom of the TAs onto cork and flash frozen in liquid nitrogen cooled iso-pentane.

Muscle samples were sectioned as previously described [27] to obtain tissue for histology and RNA analysis from defined levels within the muscle. Muscle sections were stained with haematoxylin and eosin, and by immunofluorescence for fibre type as previously described [29]. Random fields were captured at 20× magnification using an Olympus CKX41 camera and Cell^D software (Olympus Europe).

### 2.4 RNA extraction from tissue

Muscle sections from regions adjacent to the histology samples were placed into CK-14 ceramic beaded tubes containing 500 μl of TRIzol® (Invitrogen) and homogenised with the Precellys 24 (Stretton Scientific) for 2 × 15 s cycles at 5500 rpm. The samples were centrifuged at 8000 rpm for 3 min at 4 °C and the supernatant transferred to fresh micro-centrifuge tubes and RNA extracted according to manufacturer’s instructions (Qiagen). The RNA was resuspended in 30 μl RNase-free dH_2_O and stored at −80 °C. RNA concentration was quantified using a Nanodrop™.

### 2.5 Quantitative real-time PCR (QPCR)

cDNA was synthesised from 150 ng RNA and amplified by qPCR as previously described [30]. The PCR primers used have been described previously [31]. Data was normalised to a geometric mean of ribosomal protein large P0 (RPLPO) and β2 microglobulin using the ΔΔCt method.

### 2.6 Statistical analysis

Data are presented as mean ± SEM for data with a parametric distribution and median (interquartile range) for non-parametric data. Differences were determined by *t*-test for parametric data and by Mann–Whitney test for non-parametric data. To establish differences in muscle fibre profiles data were compared by one-way ANOVA for each point. Significance was set at a 2 tailed *p* value = 0.05).

## 3 Results

### 3.1 Effect of FHL1 on SMAD reporter gene expression in response to TGFβ ligands

**Fig. 1:**
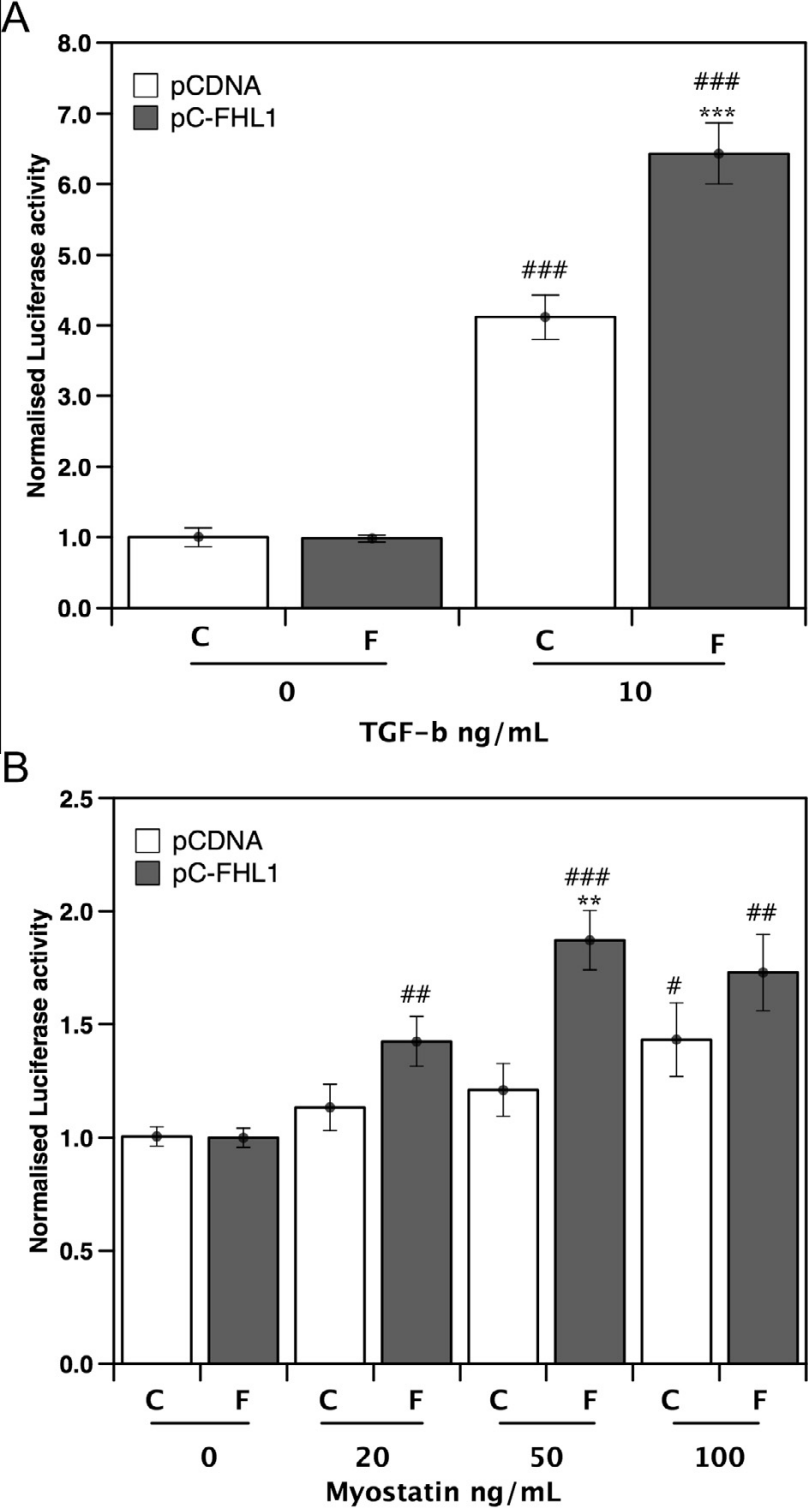
Effect of FHL1 on TGF-β ligand induced signalling. C2C12 myoblasts were transfected with a SMAD responsive luciferase reporter in the presence and absence of FHL1. Subsequently, cells were treated with either TGF-β (10 ng/mL) or myostatin at 20, 50 and 100 ng/mL. FHL1 caused a large potentiation of TGF-β induced p(CAGA)_12_ activity (A). Myostatin caused a small increase in p(CAGA)_12_ activity at 100 ng/mL. However, FHL1 enhanced p(CAGA)_12_ activity at 20 ng/mL myostatin and above (B). Data represents 4 independent experiments performed in triplicate normalised to untreated pCDNA control. ^#^*p* < 0.05, ^##^*p* < 0.01, ^###^*p* < 0.001. Compared to no myostatin for the same transfection, and ^∗∗^*p* < 0.01 FHL1 compared to control for the same myostatin concentration.

Treatment of the cells with 10 ng/mL TGFβ alone increased CAGA_12_ luciferase reporter gene expression by ∼4-fold and this was increased further to ∼7-fold by expression of FHL1 (Fig. 1a) consistent with an effect of FHL1 on TGF-β signalling. FHL1 expression had no effect on luciferase activity in the absence of added TGF-β. In the absence of transfection with an FHL1 expression plasmid (i.e. in the cells transfected with pCDNA), myostatin had no effect on luciferase activity at concentrations below 100 ng/mL. At 100 ng/mL myostatin alone increased luciferase activity ∼1.4-fold (*p* = 0.025). However, in the presence of FHL1, myostatin caused a detectable increase in luciferase activity at all concentrations above 20 ng/mL (20 ng/mL 1.4-fold, *p* = 0.003, 50 ng/mL, 1.8-fold *p* < 0.001, 100 ng/mL 1.7-fold *p* = 0.001). Furthermore, at all doses myostatin caused a larger increase in mean luciferase activity in the presence of FHL1 than in its absence (Fig. 1b) which reached statistical significance for 50 ng/mL (20 ng/mL 1.3-fold, *p* = 0.065, 50 ng/mL, 1.5-fold *p* = 0.001, 100 ng/mL 1.2-fold *p* = 0.221). These data show that FHL1 increases the activity of myostatin signalling in myoblasts. The lack of a significant effect of FHL1 on luciferase activity at 100 ng/mL myostatin raises the possibility that FHL1 increases the sensitivity of the cell to myostatin rather than the size of the response. Alternatively it may reflect the weakness of the response of the reporter system to myostatin compared to TGF-β.

### 3.2 Effect of FHL1 on myotube diameter in response to myostatin

**Fig. 2:**
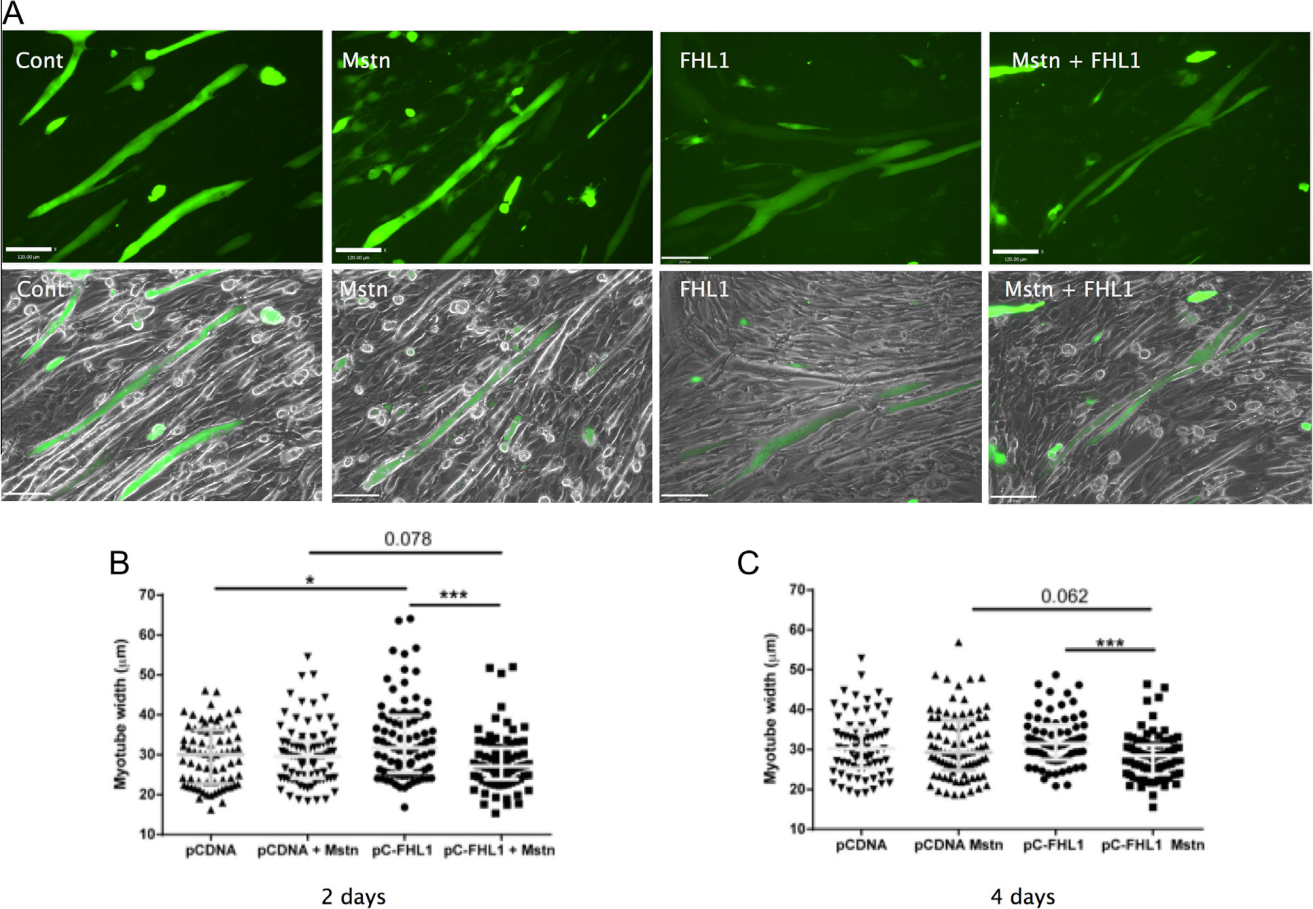
FHL1 enhances myostatin induced wasting in C2C12 myotubes. C2C12 myoblasts were co-transfected with an EGFP expression vector (pCAGGS-EGFP) and either pCDNA or pCAGGS-FHL1 and differentiated into myotubes before treatment with myostatin for 4 days. FHL1 expression induced myotube hypertrophy. At 20 ng/mL, myostatin alone had no effect on myotube diameter, however, in the presence of FHL1, myostatin reduced myotube diameter. Representative images of each group at day 2 are shown in (A) with fluorescent images alone on the top row and merged with the brightfield images below. Quantitation of myotube diameters after 2 (B) and 4 (C) days of treatment. Images captured at ×10 magnification, scale bar represents 120 μm. Graphs represent median ± IQR of pooled data from 3 independent experiments (^∗^*p* < 0.05 and ^∗∗∗^*p* < 0.001 Mann Whitney).

To determine whether FHL1 enhanced myostatin dependent myotube wasting, we determined the effect of myostatin on myotube size in the presence or absence of FHL1 expression. Treatment of the cells with 20 ng/mL myostatin alone did not alter the size of the myotubes 2 or 4 days after treatment (2 days-myostatin 29.6 μm (24.1, 33.4) Control 30.1 μm (22.8, 36.2, Fig.2a and b), 4 days-myostatin 29.3 μm, (24.9, 37.4) Control 30.4 μm, (25.3, 36.0), Fig. 2), an effect consistent with the lack of CAGA_12_ activation seen in myoblasts at this dose. Transfection of FHL1 into C2C12 cells caused a small but significant increase in myotube size (FHL1 31.1 μm (25.5, 39.8) compared to control transfection 30.1 μm (22.8, 36.2) *p* < 0.05, Fig. 2), consistent with previously described effects [15]. In contrast to the lack of effect of myostatin on control cells, in cells expressing FHL1, 20 ng/mL myostatin caused a significant reduction in myotube size both 2 and 4 days after treatment (2 days-FHL1 31.1 μm (25.5, 39.8 μm), vs FHL1 + myostatin 27.1 μm (23.9, 31.6) *p* < 0.001, 4 days-(FHL1 31.5 μm, (27.5, 36.2), FHL1 + myostatin 28.5 μm, (23.9, 31.1) *p* < 0.001) Fig. 2).

### 3.3 Effect of FHL1 on myostatin induced muscle wasting *in vivo*

**Fig. 3:**
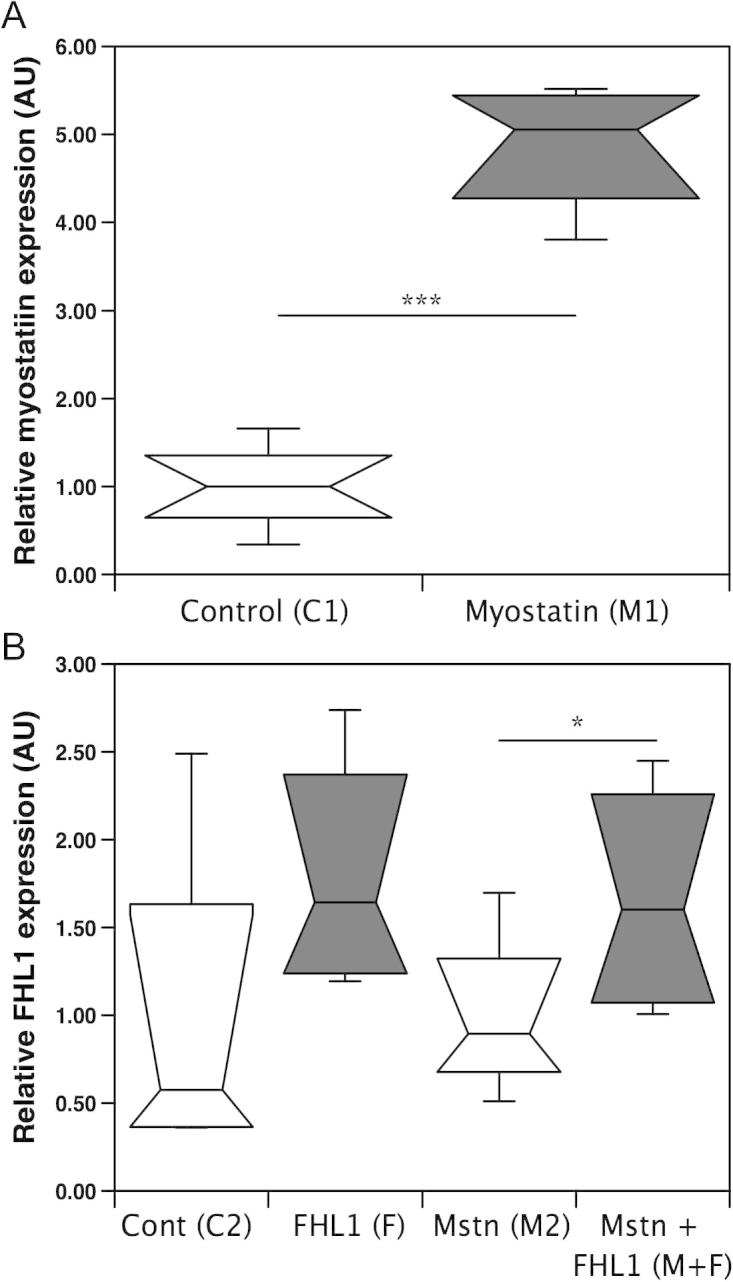
Electroporation increases myostatin and FHL1 expression *in vivo*. Expression of myostatin and FHL1 in adult mouse skeletal muscle was determined by qPCR (A and B). Myostatin expression was elevated 14 days after electroporation with pCAGGS-myostatin compared to the contralateral TA electroporated with pCAGGS alone (A). Median FHL1 expression was higher 14 days after electroporation pCAGGS-FHL1 compared to pCAGGS but this increase did not reach significance (*p* = 0.08). There was a significant increase in the FHL1 expression 14 days after electroporation with both pCAGGS-FHL1 and pCAGGS-myostatin than with pCAGGS-myostatin alone (B).

To determine whether FHL1 also increased myostatin dependent atrophy *in vivo*, we over-expressed myostatin and FHL1, alone or in combination in the *tibialis anterior* (TA) muscles of mice by electroporation. To determine the effect of myostatin or FHL1 alone, mice were injected in the right TA with pCAGGS-myostatin (M1) or pCAGGS-FHL1 (F) and in the left TA with empty pCAGGS as a control (C1 and C2 respectively). To determine the effect of FHL1 on myostatin activity, mice were electroporated in the right TA with both pCAGGS-myostatin and pCAGGS-FHL1 (M+F) and in the left TA with pCAGGS-myostatin (M2). Electroporation with pCAGGS-myostatin increased myostatin mRNA expression in M1 compared to the contralateral C1 (Fig. 3A). Similarly, electroporation of pCAGGS-FHL1 increased FHL1 expression in F and F+M muscles compared to their respective controls (C2 and M2) but this only reached statistical significance in F+M vs M2 (Fig. 3B). However, there was no effect of myostatin on FHL1 expression and FHL1 mRNA was significantly higher in the F and F+M groups combined than in all other groups combined (2-fold, *p* < 0.01, Fig. 3B). Myostatin expression in the muscle electroporated with both plasmids (F+M) did not differ from that in the contralateral muscle electroporated with pCAGGS-myostatin alone (M2) (Table 3).

**Fig. 4:**
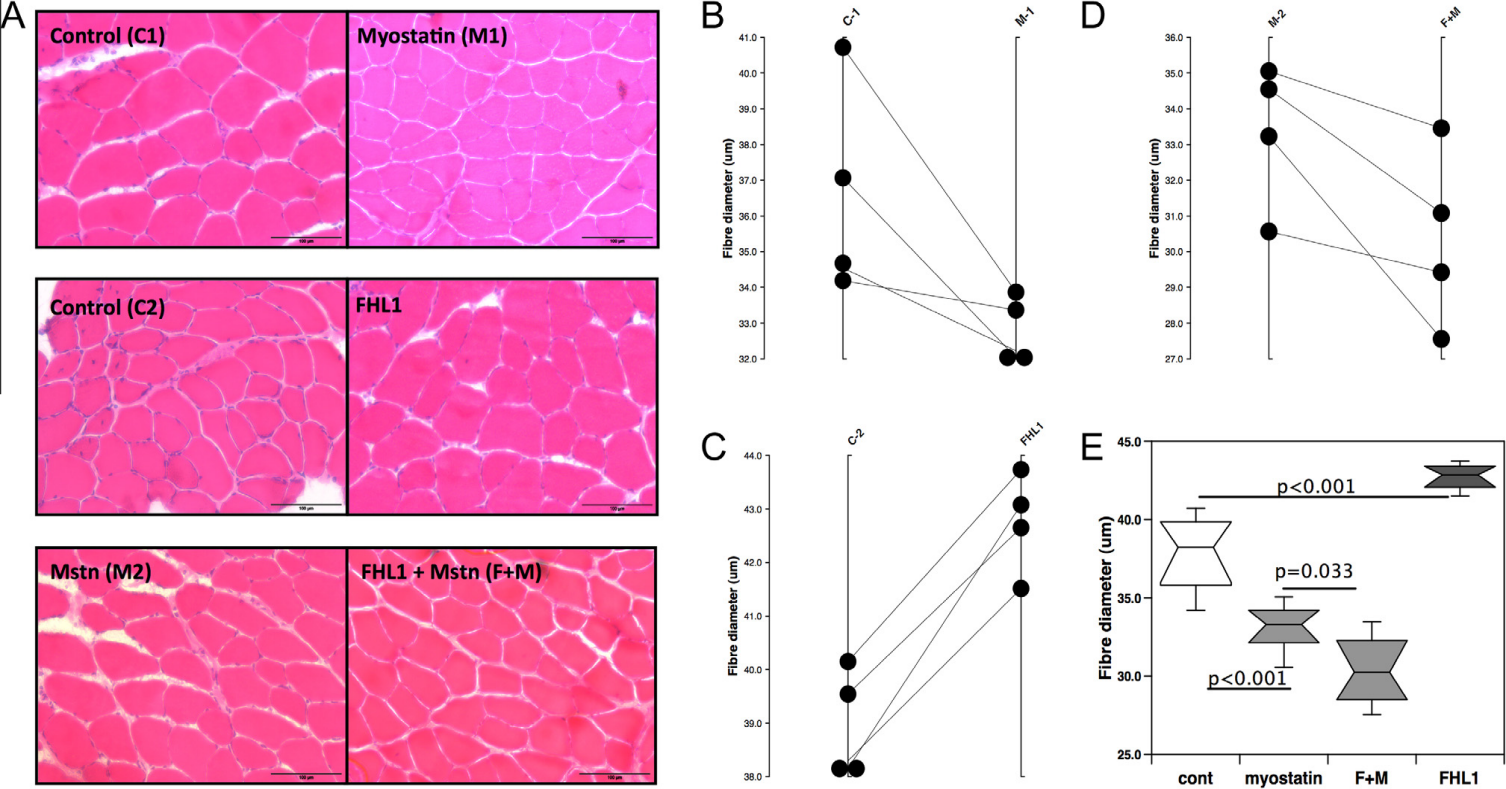
Effect of myostatin and FHL1 expression on muscle fibre size *in vivo*. Sections of the tibialis anterior were cut from the same level of each muscle and stained with haematoxylin and eosin stained sections. Representative sections are shown in (A). Comparison of fibre size in the TAs of individual mice electroporated with (B) right TA pCAGGS-myostatin (M1) and left TA pCAGGS (C1), (C) right TA pCAGGS-FHL1 (F) and left TA pCAGGS (C2), (D) right TA pCAGGS-myostatin and pCAGGS-FHL1(M+F), left TA pCAGGS-myostatin (M2). (E) unpaired analysis of fibre diameter in TAs electroporated with control vector (*n* = 8), myostatin (*n* = 8), FHL1 and myostatin (F+M, *n* = 4), FHL1 (*n* = 4) showed that myostatin reduced fibre size compared to control (*p* < 0.001), FHL1 increased fibre size compared to control (*p* < 0.001) but FHL1 + myostatin caused a larger reduction in fibre diameter than myostatin alone (*p* < 0.001 FHL1 vs FHL1 + myostatin, *p* = 0.033 myostatin vs FHL1 + myostatin). ^∗∗∗^*p* < 0.001, ^∗^*p* < 0.05.

Sections from each electroporated muscle were stained with H&E to analyse the effect of electroporation on muscle fibre diameter (Fig 4). Consistent with previous studies, myostatin expression alone caused a ∼10% decrease in fibre diameter [32] (Fig. 4A and F, from 37.8 ± 0.9 μm (*n* = 8) to 33.1 ± 0.9 μm (*n* = 8) *p* < 0.001) whereas FHL1 expression increased fibre diameter by 10% compared with the control TA (Fig. 4B and F to 42.7 ± 0.5 μm (*n* = 4) *p* < 0.001). Co-expression of FHL1 with myostatin in the same TA caused a larger decrease in fibre diameter than expression of myostatin alone (8.7% further reduction, to 30 ± 1.2 μm (*n* = 4), Fig. 4C and F, *p* = 0.033). Comparing the diameter of myofibres overexpressing FHL1 in the presence or absence of myostatin showed that myostatin decreased fibre diameter from 42.7 ± 5 μm to 30 ± 1.2 μm a reduction of approximately 25% (*p* < 0.001).

**Fig. 5:**
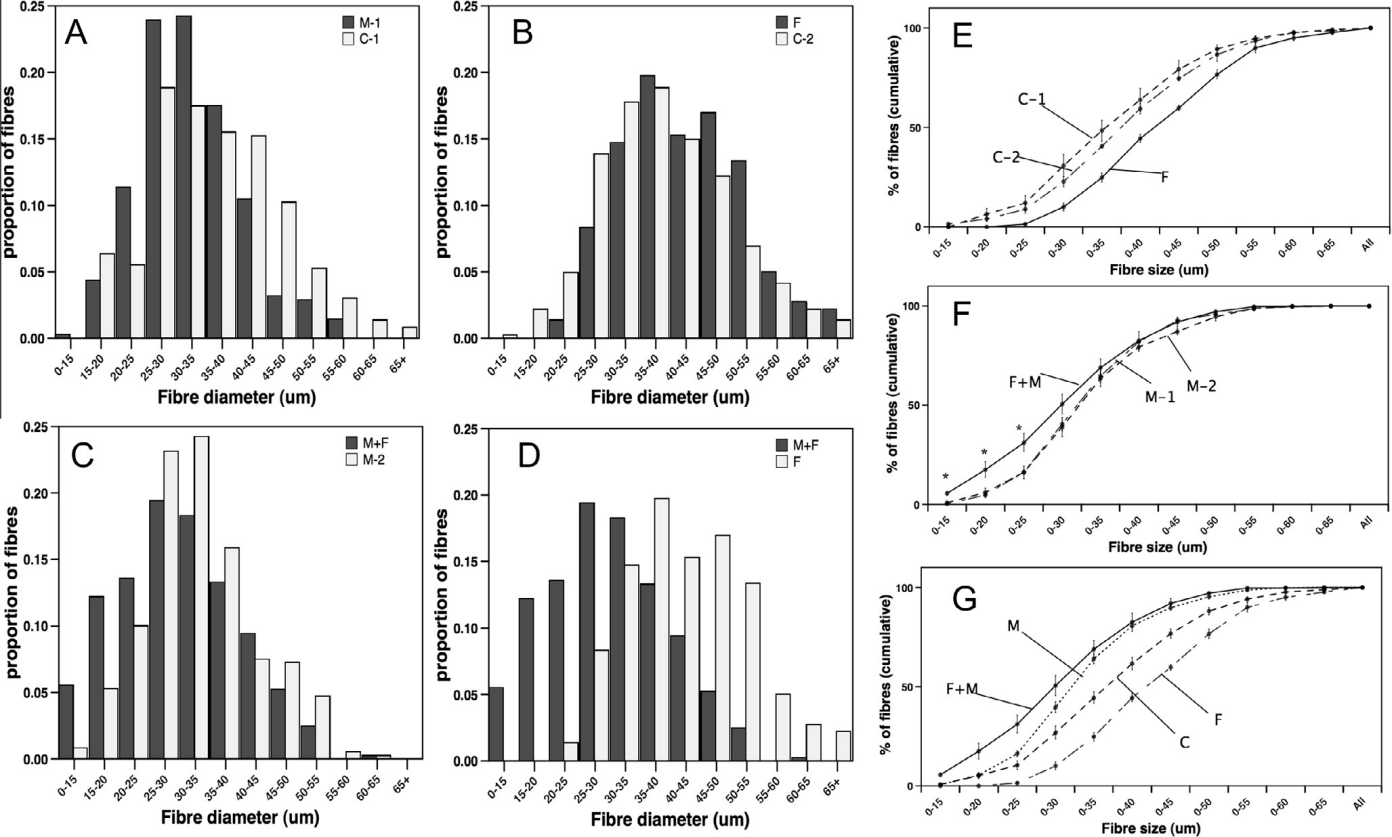
Effect of FHL1 on myostatin induced reduction in fibre size. Fibre profiles for electroporated muscles produced by determining the number of fibres from 0 to 15 μm, between 15 and 65 μm in 5 μm in bins and above 65 μm comparing left and right TA in the same animals (A–C) or between muscles expressing FHL1 alone and muscles expressing FHL1 and myostatin (D). Myostatin expression increased the proportion of small diameter fibres in the TA of mice (M-1 myostatin, C-1 control electroporated TAs from the same animals, *n* = 4), A). Electroporation of FHL1 increased the proportion of larger diameter fibres (F, FHL1 expression, C-2 control from the same animals (*n* = 4), B). Electroporation of FHL1 and myostatin together caused a greater reduction in fibre diameter than expression of myostatin alone (M+F; myostatin and FHL1 M-2; myostatin expression in the contralateral TA of the same animals (*n* = 4), C). Myostatin caused a larger decrease in fibre diameter in TAs expressing FHL1 than in those not expressing FHL1 (compare D and A). (E and G) Fibre profiles plotted as proportion of fibres below the indicated fibres diameter in 5 μm increments. E, Fibre profiles for pCAGGS electroporated muscles (C1 and C2) and for the FHL1 expressing muscle (F, the contralateral TA to C2). F. Fibre profiles for myostatin expressing muscles (M1 and M2) and the FHL1 and myostatin expressing muscle (F+M). ^∗^F+M significantly greater that M1 or M2 *p* < 0.015 ANOVA). G Fibre profiles for all muscles with data from C1 and C2 pooled (C), M1 and M2 pooled (M). Statistical analysis by ANOVA showed F+M different to M *p* < 0.001 up to 0–25 μm, M different to C at all points from 30 to 65 μm *p* < 0.003, C different to F at all points from 30 to 65 μm. Data are presented as mean ± SEM. In each group, 80–90 randomly selected fibres were measured from 8 to 9 random selected fields of view from H and E stained TA cross sections.

To analyse further the effects on muscle fibre size, we determined the proportion of fibres within 5 μm bins and the proportion of fibres below a given fibre diameter again in 5 μm steps (Fig. 5). Comparison of the two sets of control muscles with each other and the two sets of myostatin muscles with each other, showed no significant difference at any point, validating the technique and showing that expression of myostatin or FHL1 in the contralateral TA did not affect fibre size (Fig. 5E and F). This approach also allowed us to pool the data from the control and the data from the myostatin treated muscles. Myostatin caused a significant shift to smaller fibres compared to control electroporated muscles (Fig. 5A and G). FHL1 caused a significant increase in the proportion of larger fibres compared to control fibres. (Fig. 5B, E and G). In FHL1 expressing muscles, myostatin caused a greater increase in the smallest fibres than myostatin alone with a significant increase in the fibres below 25 μm (Fig 5C, F and G).

**Fig. 6:**
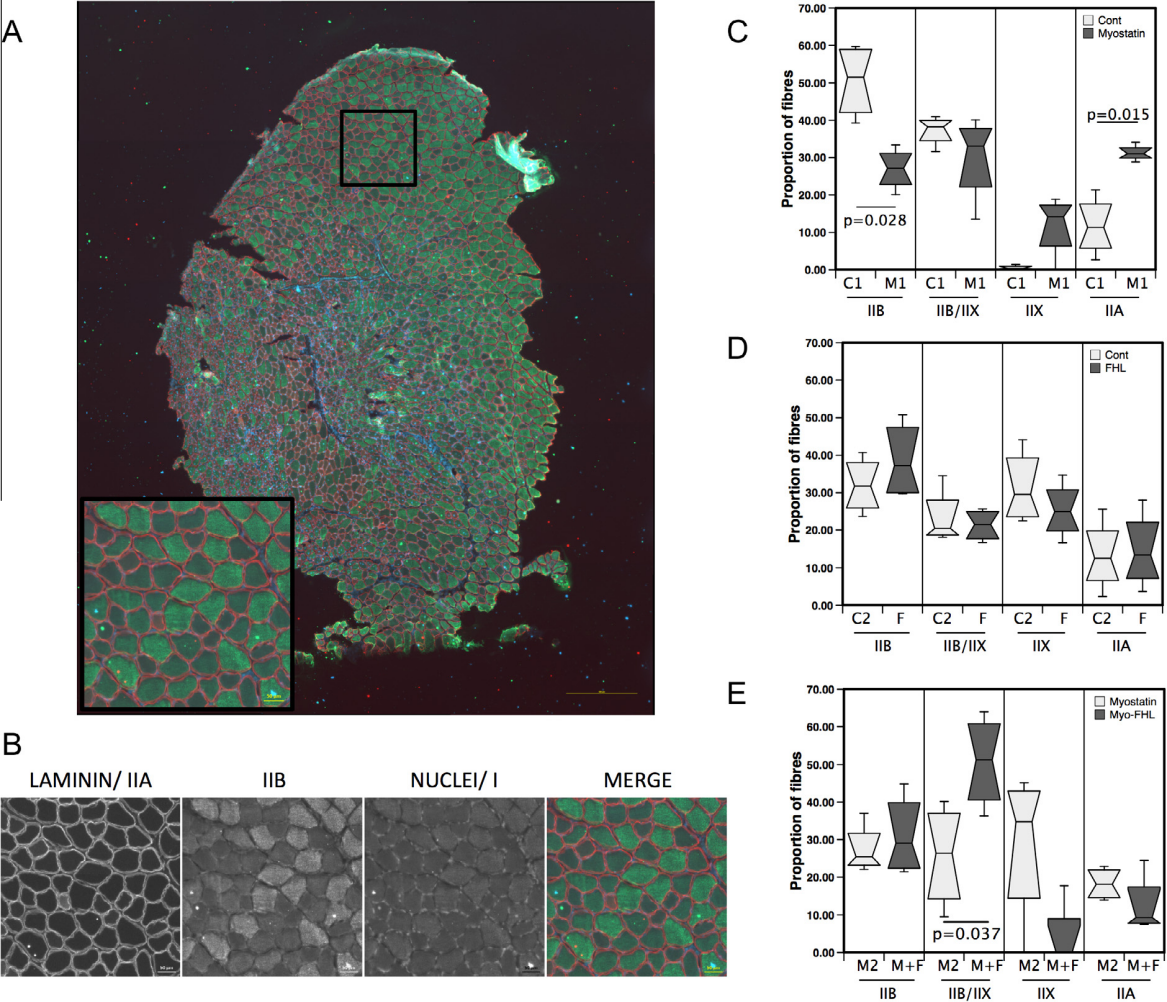
Effect of FHL1 and myostatin on fibre proportions in the TA. Sections of TA were analysed by immunofluorescence to determine the effect of FHL1 and myostatin on fibre proportions. (A) representative staining of a whole TA cross section imaged at 10x and tiled. Red = type IIA/Laminin, no stai*n* = type IIX, gree*n* = type IIB blue = type I/nuclei, scale bar = 500 μm. Region in the square is shown expanded scale bar = 50 μm. (B) individual fluorescent channels for the inset square. Fibre type analysis comparing (C) control (C1) and myostatin (M1), (D) control (C2 and FHL1 (F) and (E) myostatin (M2) and myostatin + FHL1 (M+F). Statistics show significant differences by paired *t*-test.

**Fig. 7:**
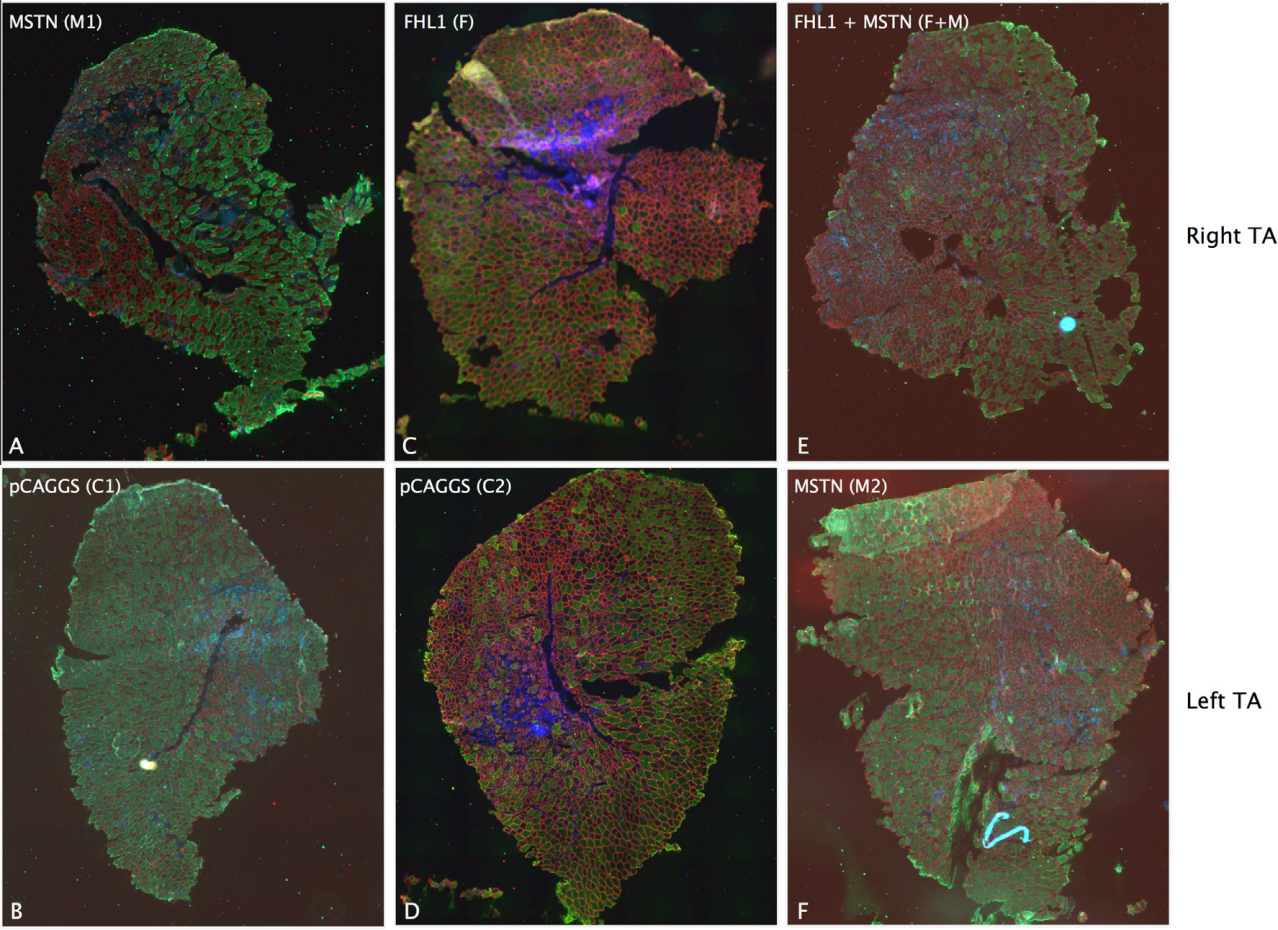
Representative images of fibre type staining. Sections of TA were analysed by immunofluorescence to determine the effect of FHL1 and myostatin on fibre proportions. The pairs of sections shown (A and B, C and D, E and F) are the right TA (top panel) and left TA (bottom panel) from the same animal and were transfected with the A; pCAGGS-MSTN, B; empty pCAGGS, C; pCAGGS-FHL1, D; empty pCAGGS, E; pCAGGS-FHL1 and pCAGGS-MSTN, F pCAGGS-MSTN. Sections were imaged at 10× and tiled. Red = type IIA/Laminin, no stai*n* = type IIX, gree*n* = type IIB blue = type I/nuclei.

Fibre proportions were analysed by immunofluorescence (Figs. 6 and 7). Comparison of the proportion of fibres in each muscle showed that myostatin expression reduced the proportion of type IIB fibres and increased the proportion of IIA fibres compared to the controls. FHL1 alone did not affect the fibre proportion compared to controls and in combination with myostatin appeared to cause an increase in type IIB/type IIX fibres (*p* = 0.037, Fig. 6).

Gene expression within the groups was then examined to identify changes in pathways associated with muscle wasting. Given that myostatin over-expression alone caused wasting, downstream TGF-β signalling was analysed, revealing a significant increase in PAI-1 (1.49-fold, M1 compared to C1, *p* < 0.05, Table 1), suggesting activation of the TGF-β signalling pathway. To identify the atrophy pathway induced by the myostatin signalling, expression of components of proteasomal degradation, autophagy and apoptosis were measured. No significant differences were observed in the expression of any of these genes but there was a trend to a decrease in expression of ATG4B (0.73-fold, *p* = 0.059) and ATG12 l (0.58-fold, *p* = 0.076), genes that are associated with autophagy. Changes in the expression of the myosin heavy chains were identified; with an increase in MHCI (13.1-fold, *p* < 0.01) and a decrease in expression of MHCIIB (0.34, *p* < 0.05, consistent with the observed reduction in type IIB fibres) and a trend towards a reduction in MHCIIX (0.64, *p* = 0.06). There was no apparent increase in the expression of MHCIIA even though there was an increase in type IIA fibre proportion possibly due to the small number of samples analysed. There was also a significant increase in myogenin expression (1.82, *p* < 0.05).

Expression of FHL1 alone did not significantly alter the expression of any of the genes tested (F compared to C2, Table 2). However, there was a trend to an increase in VEGF-C (1.2-fold, *p* = 0.085). Additionally there was a trend to a decrease in BAX expression (0.81-fold, *p* = 0.075).

Expression of FHL1 and myostatin in the same muscle (F + M compared to M2, Table 3) caused a significant increase in the expression of genes associated with autophagy (ATG12 l; 1.46-fold, *p* < 0.05 with a trend to an increase in ATG4B; 1.41-fold, *p* = 0.052) and apoptosis (BAD; 1.36-fold, *p* < 0.05 and BAX; 1.29-fold, *p* < 0.01) as well as a trend to an increase in MuRF1 (1.27-fold, *p* = 0.056, Table 3) compared to the expression of myostatin alone. There were no significant differences in the expression of myosin heavy chains in between muscles expressing myostatin alone and myostatin and FHL1 but there was a trend towards an increase in the expression of MHCIIX (1.88-fold, *p* = 0.058) and MHCI (2.99-fold, *p* = 0.075).

## 4 Discussion

Our data indicate that exogenous expression of FHL1 increases myostatin activity in skeletal muscle cells *in vitro* and exacerbates muscle wasting in the presence of elevated myostatin *in vivo*. The accepted role for FHL1 is as a promoter of hypertrophy, as shown by the effects of overexpression of FHL1 in mice and postural muscle atrophy in patients with mutations in FHL1 [15,16]. In the absence of myostatin we observed an increase in both myotube and myofibre diameter in response to FHL1 consistent with the prior data. However, in the presence of myostatin we found that FHL1 enhanced the effects of myostatin both *in vitro* and *in vivo*. These data are consistent with FHL1 potentiating the effects of TGF-β as identified in hepatic carcinoma cells [14]. However, in muscle cells FHL1 did not increase SMAD signalling in the absence of a TGF-β ligand as observed by Ding et al. in tumor cells [14]. The reason for this difference is not clear but differences in cell type with different relative levels of expression or localisation of CK1δ or SMAD proteins may provide an explanation.

The activation of myostatin by FHL1 may help to explain the increase in FHL1 following sciatic nerve section [17] and the association of FHL1 with weakness in COPD patients [19] as myostatin expression increases in both situations [33,34]. Furthermore, FHL1 is expressed at higher levels in type II fibres than in type I fibres raising the possibility that it contributes to the greater sensitivity of type II fibres to myostatin.

Myostatin has previously been shown to increase the expression of the atrogenes MuRF-1 and atrogin-1 and we have previously shown that myostatin increases the expression of autophagy-associated genes *in vitro* [31,35]. Whilst there was no detectable increase in the expression of genes associated with these pathways in the muscles treated with myostatin alone in the presence of both myostatin and FHL1, the expression of genes associated with autophagy and apoptosis were increased consistent with muscle atrophy. The lack of increase in the presence of myostatin alone may have been the result of the time point studied thus any earlier increase in the expression of these genes may have been missed. Such an explanation is consistent with the small increases observed in the presence of myostatin and FHL1 where the atrophy was greater.

### 4.1 Critique of the experimental approach

The data present a consistent argument that FHL1 increases the functional effects of myostatin *in vitro* and *in vivo*. This observation is also consistent with activation of SMAD signalling by FHL1 in other cells. However, there are a number of potential confounding factors in the data that need to be considered. First it should be noted that the experimental approach relies on over-expression and it is possible that this causes an artefact. Against this suggestion is the consistency of the responses in myoblasts, myotubes and muscle *in vivo* as FHL1 increases myostatin activity measured by 3 different assays (luciferase based SMAD reporter assays, myotube wasting *in vitro* and myofibre wasting *in vivo*. The second potential confounding issue is that the *in vivo* response occurs on the background of a regeneration response to electroporation injury complicating any picture. However, our observations on the effects of the single agents (myostatin and FHL1) are consistent with previous studies using transgenic over-expression, which does not cause injury and regeneration [15,36]; with myostatin alone causing atrophy and FHL1 alone causing hypertrophy. Furthermore, the effects we observe *in vitro* are the same as those we observe *in vivo* (i.e. FHL1 alone increases myotube size whereas in combination with myostatin it causes a greater reduction in myotube size than myostatin alone). Together these observations suggest that the combined response *in vivo* is not caused by the experimental approach. The third confounding factor is that we did not observe large changes in gene expression in response to either FHL1 or myostatin. Indeed only the increase in myostatin (which we over expressed) and MCH-I exceeded a 2-fold change although a number of other changes reached statistical significance. These small changes are likely to result from the time-point chosen for the analysis which is 2 weeks after the electroporation and the likely reduction in the expression of the transfected genes with time. Such small changes and the fact that many trends did not reach statistical significance may account for the apparent opposite direction of change observed for a number of the genes between myostatin compared to control (Table 1) and myostatin + FHL1 compared to myostatin (Table 3). However, as FHL1 also interacts with a number of transcription factors including NFATC1 [15] it is also possible that these differences in direction of change reflect a different time course that results from the interaction between FHL1 and myostatin or some other factor within the experimental system.

## 5 Conclusion

In conclusion we demonstrate that exogenous FHL1 expression exacerbates the atrophic effects of myostatin *in vitro* and *in vivo*. These observations together with the relative restriction of FHL1 to type II fibres observed in the literature [16], suggest that FHL1 may contribute to type II fibre atrophy under the appropriate conditions. However, further experiments are required to confirm that FHL1 contributes to the increased sensitivity of type II fibres to myostatin dependent atrophy.

## Figures and Tables

**Table 1:** Effect of Myostatin expression on muscle gene expression in the TA.

		Control (C1)		Myostatin (M1)		
Group	Gene	AV	SEM	AV	SEM	*p*
	FHL1	1.00	0.22	0.77	0.14	Ns
	myostatin	1.00	0.27	4.86	0.39	**

Myosins	MyHC1	1.00	0.28	13.1	4.49	**
	MyHC2x	1.00	0.34	0.64	0.15	0.060
	MyHC2a	1.00	0.40	1.26	0.46	Ns
	MyHC2b	1.00	0.32	0.34	0.09	*

TGF-β signalling	VEGF-C	1.00	0.25	0.80	0.14	Ns
PAI-1	1.00	0.29	1.49	0.27	*

Autophagy	ATG-4B	1.00	0.19	0.73	0.15	0.059
LC3B	1.00	0.22	0.85	0.20	Ns
ULK-2	1.00	0.27	0.67	0.14	Ns
ATG-12	1.00	0.32	0.58	0.13	0.076

Apoptosis	Bad	1.00	0.18	0.92	0.15	Ns
Bax	1.00	0.18	0.84	0.09	Ns

Atrophy	Atrogin	1.00	0.16	0.75	0.15	Ns
Murf	1.00	0.21	0.81	0.17	Ns

Myogenesis	MyoD	1.00	0.30	1.04	0.21	Ns
Myogenin	1.00	0.24	1.82	0.27	*

Table of fold changes for each gene with expression in the myostatin expressing muscle (M1) compared to expression in the contralateral controls (C1). Values are normalised to average of the control TAs (C1) group (*n* = 4 in each group). Data represent mean ± SEM, where ^∗^*p* < 0.05, ^∗∗^*p* < 0.01 from paired *t*-test of log transformed data.

**Table 2:** Effect of FHL1 expression on muscle gene expression in the TA.

		Control (C2)		FHL1 (F)		
Group	Gene	AV	SEM	AV	SEM	*p*
	FHL1	1.00	0.51	1.80	0.36	0.083
	Myostatin					

Myosins	MyHC1	1.00	0.59	0.61	0.23	Ns
	MyHC2x	1.00	0.44	1.62	0.23	Ns
	MyHC2a	1.00	0.60	1.26	0.41	Ns
	MyHC2b	1.00	0.48	1.51	0.28	Ns

TGF-β signalling	VEGF-C	1.00	0.40	1.20	0.25	Ns
PAI-1	1.00	0.35	0.99	0.13	Ns

Autophagy	ATG-4B	1.00	0.37	0.83	0.17	Ns
LC3B	1.00	0.40	0.91	0.20	Ns
ULK-2	1.00	0.38	0.80	0.21	Ns
ATG-12	1.00	0.36	1.16	0.12	Ns

Apoptosis	Bad	1.00	0.54	0.79	0.14	Ns
Bax	1.00	0.10	0.81	0.20	0.075

Atrophy	Atrogin	1.00	0.43	0.88	0.11	Ns
Murf	1.00	0.37	0.83	0.09	Ns

Myogenesis	MyoD	1.00	0.48	0.66	0.12	Ns
Myogenin	1.00	0.27	0.76	0.02	Ns

Table of fold changes for each gene with expression in the FHL1 expressing muscle (F) compared to expression in the contralateral controls (C2). Values are normalised to average of control TAs (C2) (*n* = 4 in each group). Data represent mean ± SEM.

**Table 3:** Effect of FHL1 expression on muscle gene expression in the muscles that express myostatin.

		Myostatin (M2)		Myostatin + FHL1 (M+F)		
Group	Gene	AV	SEM	AV	SEM	*p*
	FHL1	1.00	0.25	1.66	0.35	*
	Myostatin	1.00	0.09	0.93	0.31	Ns

Myosins	MyHC1	1.00	0.56	2.99	1.14	0.075
	MyHC2x	1.00	0.09	1.88	0.44	0.058
	MyHC2a	1.00	0.25	1.45	0.47	Ns
	MyHC2b	1.00	0.29	1.24	0.52	Ns

TGF-β signalling	VEGF-C	1.00	0.21	1.23	0.17	Ns
PAI-1	1.00	0.22	1.38	0.54	Ns

Autophagy	ATG-4B	1.00	0.25	1.41	0.24	0.052
LC3B	1.00	0.21	1.15	0.27	Ns
ulk-2	1.00	0.31	1.34	0.44	Ns
ATG-12	1.00	0.15	1.46	0.22	*

Apoptosis	Bad	1.00	0.15	1.29	0.24	*
Bax	1.00	0.12	1.36	0.20	**

Atrophy	Atrogin	1.00	0.20	1.23	0.19	Ns
Murf	1.00	0.17	1.27	0.25	0.056

Myogenesis	MyoD	1.00	0.23	1.25	0.22	Ns
Myogenin	1.00	0.31	0.85	0.16	Ns

Table of fold changes for each gene with expression in the myostatin + FHL1 muscles (M+F) expressing muscle compared to expression in the contralateral myostatin expressing muscles (M2). Values are normalised to the average of the M2 TAs (*n* = 4 in each group). Data represent mean ± SEM, where ^∗^*p* < 0.05, ^∗∗^*p* < 0.01 from paired *t*-test of log transformed data.
